# Repeated lumbar punctures within 3 days may affect CSF biomarker levels

**DOI:** 10.1186/s12987-019-0157-2

**Published:** 2019-12-13

**Authors:** Martin Olsson, Johan Ärlig, Jan Hedner, Kaj Blennow, Henrik Zetterberg

**Affiliations:** 1 Clinical Neurochemistry Laboratory, Sahlgrenska University Hospital, House V, 431 80 Mölndal, Sweden; 2 Department of Psychiatry and Neurochemistry, Institute of Neuroscience and Physiology, University of Gothenburg, Mölndal, Sweden; 3 Center for Sleep and Vigilance Disorders, Institute of Medicine, University of Gothenburg, Gothenburg, Sweden; 4 Department of Anaesthesiology and Intensive Care Medicine, Sahlgrenska University Hospital/Östra, Gothenburg, Sweden; 5 Sleep Laboratory, Department of Pulmonary Medicine, Sahlgrenska University Hospital, Gothenburg, Sweden; 6 Department of Neurodegenerative Disease, UCL Institute of Neurology, Queen Square, London, UK; 7 UK Dementia Research Institute at UCL, London, UK

**Keywords:** Lumbar puncture, Cerebrospinal fluid, Alzheimer’s disease, Biomarkers, Amyloid β, Tau, GFAp, Neurofilament light, YKL-40

## Abstract

Lumbar puncture (LP) is a common way of collecting cerebrospinal fluid (CSF) both in the clinic and in research. In this extension of a study on the relationship between sleep deprivation and CSF biomarkers for Alzheimer’s disease, we investigated CSF biomarker dynamics in relation to rebound sleep after sleep deprivation. Two LPs were performed within 3 days in 13 healthy volunteers. We noticed an unexpected sharp rise in biomarker concentrations in the second sample and therefore repeated the experiment, but without sleep intervention, in four additional individuals. The findings were similar in these subjects, suggesting an inherent methodological problem with repeated LPs. The result corroborates findings in studies with repeated CSF collection via indwelling lumbar catheters, and needs to be addressed in, for instance, pharmacodynamic studies employing these techniques.

## Introduction

Lumbar puncture (LP) is a routine procedure used in the clinical setting, as well as in research, to collect cerebrospinal fluid (CSF). A wide range of biomarkers can be measured in CSF, making LP a useful procedure in diagnostics of and research on traumatic injuries, infections, autoimmune conditions, bleeding and other CNS pathologies. More specifically, LP has emerged as a key tool in the diagnosis of neurodegenerative disease [1].

We have previously examined the link between partial sleep deprivation and biomarkers for Alzheimer’s disease (AD) in both CSF and plasma [2, 3]. In that research setting, with repeat LPs, we also observed a marked increase in several, but not all, brain biomarkers in the sample tapped at the second LP taken 3 days later. This was in consistency with the continuous increase in CSF Aβ levels reported during repeat CSF sampling via an indwelling lumbar catheter during 36 h [4]. In this paper, we report on these findings and discuss possible mechanisms.

## Materials and methods

In a previously published study [2], 13 healthy adults (Table 1) with normal sleeping habits were exposed to five nights of controlled normal sleep and five nights of restricted sleep (< 4 h of sleep). LP was performed in the morning following each period. In an ad-hoc experiment on recovery sleep, another LP was performed 72 h after the post-sleep deprivation LP. The participants were allowed to sleep freely during this period. Caffeine, nicotine or other central stimulating agents were not allowed. Because of a suspected artefact the data from the ad-hoc data was not published, but instead, a follow-up study was planned.

**Table 1:** Anthropometric and baseline data

Variable	Mean (SD)	
	Recovery sleep cohort, N = 13^a^	Repeated LP cohort, N = 4
Anthropometric variable		
Age, years	25 (4.0)	25 (1.3)
Weight, kg	79.3 (13.6)	75.8 (12.1)
Height, cm	184.2 (14.0)	181.0 (16.1)
BMI, kg/m^2^	23.4 (2.4)	23.3 (3.3)
Pulse, bpm	60 (6)	67 (9)
Systolic BP, mmHg	134 (5)	128 (9)
Diastolic BP, mmHg	81 (6)	80 (6)
ESS	6 (3)	4.8 (3.3)
Baseline characteristics	No (%)	
Gender, male	9 (69.2)	2 (50)
Nicotine, smoker	3 (23.1)	0 (0.0)
Alcohol, > 15 standard units	1 (6.2)	0 (0.0)

*BMI* body mass index, *BP* blood pressure, *ESS* Epworth Sleepiness Scale

^a^Previously published data [2]

**Fig. 1:**
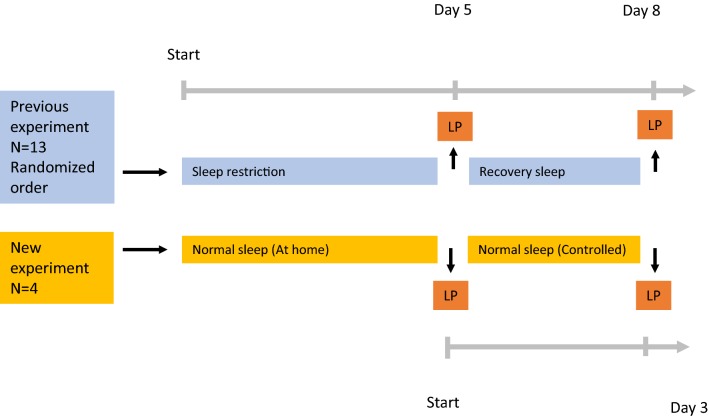
Study flowchart. *LP* lumbar puncture

In the follow-up experiment, four healthy adults (Table 1) with the same inclusion and exclusion criteria as in the original study were subjected to two consecutive LPs in the morning, 3 days apart and without any other intervention. Sleep was monitored by actigraphy (Fig. 1).

### CSF sampling and analysis

CSF samples were collected by LP at the L3/L4 or L4/L5 interspace with a 22gx90mm Sprotte™ needle, by an experienced neurologist. The needle type was chosen to minimize the risk of post-LP headache [5]. Sampling was performed between 8 and 9 am 3 days apart. A total of 10–12 mL of CSF was collected in polypropylene tubes, centrifuged at 1300*g* for 10 min, aliquoted and stored in 0.5 mL aliquots at − 80 °C pending analysis within 1 h after sampling.

CSF Aβ38, Aβ40 and Aβ42 concentrations were measured using MSD Abeta Triplex (Meso Scale Discovery, Rockville, Maryland). CSF total tau (T-tau) and phosphorylated tau (P-tau) concentrations were measured using INNOTEST sandwich enzyme-linked immunosorbent assays (ELISAs, Fujirebio, Ghent, Belgium). CSF neurofilament light (NF-L) concentration was measured using the NF-Light ELISA (UmanDiagnostics, Umeå, Sweden). CSF YKL-40 (also called chitinase 3-like 1) concentration was measured using the Human Chitinase 3-like 1 Quantikine ELISA Kit (R&D Systems, Inc. Minneapolis, MN). CSF glial fibrillary acidic protein (GFAP) concentration was measured using an in-house ELISA [6]. All measurements were performed in one round of experiments with one batch of reagents and baseline and follow-up samples side by side on the assay plates by board-certified laboratory technicians who were blinded to clinical data.

### Sleep surveillance

ActiGraph GT3X+ devices were worn on the nondominant wrist, throughout the experiment. Data from the devices was used to validate a normal sleep pattern with at least 8 h of bedtime. Actigraphy data was reviewed with the ActiLife software and analyzed with the Sadeh algorithm [7, 8].

### Statistics

Statistical analysis was done using IBM SPSS version 25.0. Significance was calculated by two-tailed paired sample t-test and alpha was set to 0.05.

## Results

**Fig. 2:**
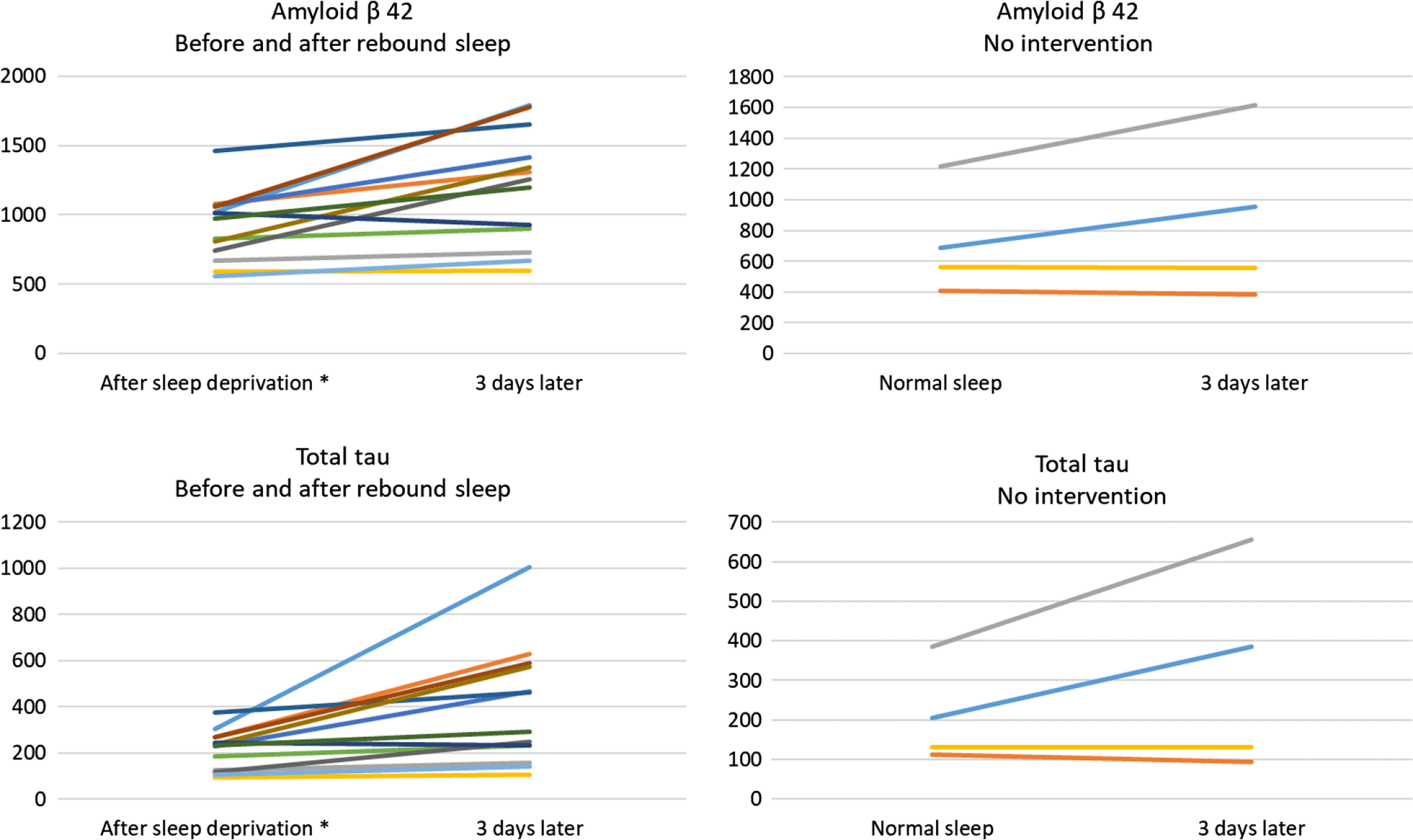
Individual concentration changes. Individual CSF biomarker concentration changes between first and second lumbar puncture. All samples are taken 3 days apart. All concentrations are in pg/mL. *Previously published data [2]

In conjunction with our earlier experiment, we investigated the effect of recovery sleep on a set of AD biomarkers (Fig. 1). An unexpectedly sharp increase in CSF concentrations of several AD biomarkers after recovery sleep was observed in the sample collected 3 days after the baseline LP (Fig. 2, Table 2). A similar, but less sharp increase in CSF biomarker concentrations was evident in CSF obtained 3 days after a first LP following normal sleep exposure. In contrast, NF-L and GFAP did not change significantly in either the original or the ad hoc protocol.

**Table 2:** CSF biomarker concentrations

	Recovery sleep				No sleep intervention			
	LP 1 x̄ ^a^	LP 2 x̄	FC	p	LP 1 x̄	LP 2 x̄	FC	p
NFL	325	305	0.94	0.231	198	194	0.98	N/A
GFAp	183	188	1.03	0.641	236	231	0.98	N/A
T-tau	215	395	1.83	0.008	208	317	1.52	N/A
P-tau	39	59	1.51	0.006	31	43	1.39	N/A
Ab38	2640	3611	1.37	0.005	2353	2930	1.25	N/A
Ab40	7432	9434	1.27	0.006	6249	7117	1.14	N/A
Ab42	913	1198	1.31	0.003	719	878	1.22	N/A
YKL-40	56,718	72,363	1.28	0.039	56,978	71,639	1.26	N/A

LP 1 x̄ and LP 2 x̄ represent average absolute concentrations in the first and second lumbar puncture, measured in pg/mL. p represents probability value calculated by paired two-tailed t-test

*YKL-40* chitinase-3-like protein, *NFL* neurofilament light, *GFAp* glial fibrillary acidic protein, *T Tau* total tau. *P-tau* phosphorylated tau *FC* fold change of each biomarker

^a^Previously published data [2]

## Discussion

The rise in the concentration of several AD biomarkers suggests an inherent methodological problem with repeated LPs within a few days. Studies analyzing repeat CSF samples taken through an indwelling lumbar catheter also report marked increases in Aβ levels, up to > 100% of baseline levels during the first 12 h, and then fluctuating around double baseline levels for 36 h [4]. A plausible explanation is that the large volume of CSF tapped (in total 6 × 36 = 216 mL) may cause a disturbance in the CSF dynamics (flow of CSF from the cerebrum down to the lumbar sac), as suggested by a study showing that a lower CSF sampling frequency (and thus lower CSF volume tapped) reduced such effects on Aβ levels [9]. Alternative explanations include that CSF levels of brain biomarkers are not homogeneously distributed within the CSF space. Among the tested peptides, Aβ (all isoforms) and tau (both total and phosphorylated) have particularly high expression in the cerebrum, as opposed to NFL and GFAp, the expression of which is more evenly distributed throughout the CNS. With high volumes of CSF tapped from the lumbar region, redistribution of CSF originating from regions with close proximity to the cortex to the lumbar sac [9] may occur.

In the present study, we found similar, but less pronounced (only 12 mL of CSF was tapped at the first LP) increases in CSF levels of brain biomarkers, which may have similar explanations. Contributing explanations may be CSF leakage after the LP into the under-pressured epidural space; an explanation supported by the finding that subjects with post-procedure headache (that is known to be caused by CSF leakage) had a more marked increase in CSF concentrations of Aβ than those without this complication [9]. A limitation of the present study is the small number of study objects making significance testing less meaningful. Further, better powered, studies specifically investigating the proposed effect are thus needed.

We would now like to alert the research community of an apparent effect of repeated LPs on CSF biomarker concentrations. It is also worth noting that we used a “non-traumatic” needle type. Using standard needles might have aggravated the biomarker changes even further. These results are important to consider when designing studies with repeated LPs. They may also be of relevance in studies where spinal catheters are placed to examine dynamic changes in CSF composition.

## Conclusion

An LP in itself may have profound effects on CSF protein concentrations if samples are collected by repeated LPs 3 days apart. This confounding influence needs to be taken into account in CSF biomarkers studies with repeat LPs, such as pharmacodynamic Phase I trials.

## Data Availability

The datasets generated and/or analysed during the current study are available in the Swedish National Data Service (SND) repository: 10.5878/tpbk-k885, 10.5878/cavp-eh78

## Abbreviations

Aβ: amyloid β
AD: Alzheimer’s disease
CSF: cerebrospinal fluid
GFAP: glial fibrillary acidic protein
LP: lumbar puncture
NF-L: neurofilament light
